# Comparison of Splenectomy and Eltrombopag Treatment in the Second-Line Treatment of Immune Thrombocytopenic Purpura

**DOI:** 10.4274/tjh.galenos.2021.2021.0216

**Published:** 2021-08-25

**Authors:** Mehmet Can Uğur, Sinem Namdaroğlu, Esma Evrim Doğan, Esra Turan Erkek, Nihan Nizam, Rafet Eren, Oktay Bilgir

**Affiliations:** 1 University of Health Sciences Turkey, İzmir Bozyaka Training and Research Hospital, Clinic of Hematology, İzmir, Turkey; 2 University of Health Sciences Turkey, Prof. Dr. Cemil Taşçıoğlu Training and Research Hospital, Clinic of Hematology, İstanbul, Turkey; 3 University of Health Sciences Turkey, İzmir Dr. Lütfi Kırdar Training and Research Hospital, Clinic of Hematology, İstanbul, Turkey; 4 İzmir Çiğli Training and Research Hospital, Clinic of Internal Medicine, İzmir, Turkey

**Keywords:** Thrombocytopenia, Eltrombopag, Splenectomy

## Abstract

**Objective:**

Primary immune thrombocytopenia (ITP) is an acquired autoimmune disease characterized by isolated thrombocytopenia. While first-line treatments focus on inhibiting autoantibodies and platelet destruction, second- and third-line treatments include splenectomy and thrombopoietin receptor agonists. In this study, we aimed to compare the efficiency and toxicities of splenectomy and eltrombopag as second-line treatments in ITP.

**Materials and Methods:**

We retrospectively analyzed patients who were diagnosed with ITP and followed between 2015 and 2020. Patients who underwent splenectomy or received eltrombopag treatment as second-line or further therapy were included. For subgroup analyses, patients were further stratified according to whether they received eltrombopag in the second or third line of treatment.

**Results:**

There were 38 patients in the splenectomy group and 47 patients in the eltrombopag group. The mean age of patients in the splenectomy and eltrombopag groups was 43.2 and 50.5 years, respectively. Time to response was significantly shorter in the splenectomy arm (p=0.001). However, response rates at the 3^rd^, 6^th^, 12^th^, and 24^th^ months did not exhibit a statistically significant difference between groups; nor did total duration of response and adverse events. Response rates at the 1^st^, 3^rd^, 6^th^, 12^th^, and 24^th^ months and the total duration of response did not exhibit a statistically significant difference between eltrombopag subgroups. Eltrombopag treatment was ceased for 20 patients after a median of 54.1 months (range: 1-151). Among them, 12 patients (60%) did not experience a loss of response.

**Conclusion:**

Comparing the splenectomy and eltrombopag arms, even though time to achieve response was in favor of the splenectomy group, this advantage disappeared when overall response rates and response rate at the 2^nd^ year were considered. Using eltrombopag in the second or third line of therapy does not yield any difference in terms of time to achieving response.

## Abstract (tr)

**Amaç:**

Primer immün trombositopeni (İTP), izole trombositopeni ile karakterize, edinsel bir otoimmün hastalıktır. Birinci basamak tedaviler otoantikorları ve trombosit yıkımını inhibe etmeye odaklanırken, ikinci ve üçüncü basamak tedaviler arasında splenektomi ve trombopoietin reseptör agonistleri bulunur. Bu çalışmada, İTP’de ikinci basamak tedaviler olarak splenektomi ve eltrombopagın etkinlik ve toksisitelerinin karşılaştırılması amaçlanmıştır.

**Gereç ve Yöntemler:**

2015-2020 yılları arasında İTP tanısı alan ve takip edilen hastaları retrospektif olarak analiz ettik. Splenektomi yapılan ve 2. veya daha ileri basamaklarda eltrombopag tedavisi alan hastalar çalışmaya dahil edildi. Alt grup analizleri için, hastalar ikinci veya üçüncü tedavi hattında eltrombopag alıp almadıklarına göre gruplandırıldı.

**Bulgular:**

Splenektomi grubunda 38 hasta, eltrombopag grubunda 47 hasta vardı. Splenektomi ve eltrombopag gruplarındaki hastaların ortalama yaşı sırasıyla 43,2 ve 50,5’di. Splenektomi kolunda yanıt süresi anlamlı olarak daha kısaydı (p=0,001). Bununla birlikte, 3., 6., 12. ve 24. aylardaki yanıt oranları, toplam yanıt süresi ve yan etkiler açısından gruplar arasında istatistiksel olarak anlamlı fark bulunmadı. Birinci, 3., 6., 12. ve 24. aylardaki yanıt oranları ve toplam yanıt süresi, eltrombopag alt grupları arasında istatistiksel olarak anlamlı değildi. Eltrombopag tedavisi medyan 54,1 ay (1-151) sonra 20 hastada kesildi. Bunlardan 12 hastada (%60) yanıt kaybı yaşanmadı.

**Sonuç:**

Splenektomi ve eltrombopag kolları karşılaştırıldığında yanıt alma süresi splenektomi lehine olsa da, genel yanıt oranları ve 2. yıldaki yanıt oranları dikkate alındığında bu avantaj ortadan kalkmaktadır. İkinci veya 3. basamak tedavide eltrombopag kullanılması, yanıt alma süresi açısından herhangi bir fark oluşturmamaktadır.

## Introduction

Primary immune thrombocytopenia (ITP) is an acquired autoimmune disease characterized by isolated thrombocytopenia caused by T-cell-mediated platelet destruction, with immunoglobulin G autoantibodies binding to platelets and megakaryocytes, and megakaryocyte dysfunction. Patients may present with petechiae, purpura, mucosal bleeding, and life-threatening organ bleeding [1,2,3]. The aim of treatment is to prevent serious or life-threatening bleeding. Treatment modalities employed in ITP target various stages in its pathophysiology, including the inhibition of autoantibody synthesis, modulation of T-cell activity, and stimulation of platelet production. While first-line treatments mainly focus on inhibiting autoantibodies and platelet destruction, second- and third-line treatments include immunosuppression, splenectomy, and megakaryocyte stimulation for increased platelet production [4].

Splenectomy is an effective treatment choice for steroid-refractory or steroid-dependent ITP because the spleen is the major site of platelet clearance. Macrophages express the FcγR function in the phagocytosis of antibody-coated platelets via SYK signaling pathways [5,6]. They also present antigenic peptides, including glycoproteins IIa/IIIb and Ib/IX, to CD4+ T cells, causing activation and expansion of autoreactive B- and T-cells [7,8]. The spleen also acts as a reservoir for long-lived plasma cells producing antiplatelet antibodies [9]. However, there is no reliable predictor for splenectomy response. Considering the associated risk of infection and cardiovascular complications, splenectomy is being replaced by immunosuppressive agents and thrombopoietin receptor agonists (TPO-RAs) as the second-line treatment of ITP [10].

The TPO-RAs romiplostim and eltrombopag have been widely used since 2008 [11]. These agents bind to the TPO receptor, cause conformational changes, and activate the JAK2/STAT5 pathway, increasing megakaryocyte progenitor proliferation and platelet production [12,13]. Randomized controlled studies with TPO-RAs have reported response rates of 50% to 90%, as well as efficiency in preventing bleeding and decreased need to use additional medication. Data on toxicity indicate reversible reticulin fibrosis and increased risk of venous thromboembolism (VTE) [11].

Studies that directly compare the long-term efficacy of second-line treatments for ITP are limited and treatment decisions are therefore mostly patient-based. In this study, we aimed to compare the efficiency and toxicities of splenectomy and eltrombopag, a TPO-RA, as second-line treatments for ITP.

## Materials and Methods

In our study, we retrospectively analyzed patients who were diagnosed with ITP after the evaluation of complete blood count, peripheral blood smear, blood biochemistry, viral serologies including human immunodeficiency virus and hepatitis C virus, abdominal sonography, and antinuclear antibody testing and who were followed between 2015 and 2020. Bone marrow aspiration and biopsy were performed for patients as deemed necessary by the clinician to confirm the diagnosis of ITP. Among these cases, patients who were steroid-resistant, who were steroid-refractory, or who experienced relapse after response and who had undergone splenectomy or received eltrombopag treatment as second-line or third-line therapy as the next step after only splenectomy were included in the study. Included subjects were divided into splenectomy and eltrombopag groups. For subgroup analyses, patients receiving eltrombopag were further stratified according to whether they received eltrombopag as the second or third line of treatment.

Patient data were recorded, including age, sex, ITP bleeding score, white blood cell (WBC) count, neutrophil and lymphocyte counts, hemoglobin level, mean corpuscular volume (MCV), platelet count, mean platelet volume (MPV), serum creatinine, aspartate aminotransferase, alanine aminotransferase, and lactate dehydrogenase levels at the time of splenectomy or initiation of eltrombopag therapy, as well as the dates of diagnosis and last visit. The ITP bleeding score was assessed according to the World Health Organization Bleeding Scale [14]. Cases were classified using a simple five-point scale in which no bleeding was scored as 0, petechiae as grade 1, mild blood loss as grade 2, gross blood loss as grade 3, and debilitating blood loss as grade 4. The following comparisons between the splenectomy and eltrombopag groups were also performed: time to achieve response; response at the 1^st^, 3^rd^, 6^th^, 12^th^, and 24^th^ months; total duration of response; and complications. Response evaluation was performed according to the American Society of Hematology’s 2009 International Working Group Report [15]. Platelet count below 30,000/mm^3^ was considered as no response, above 30,000/mm^3^ as response, and above 100,000/mm^3^ as complete response.

The compliance of the study with ethical rules was confirmed by the İzmir Bozyaka Training and Research Hospital Ethics Committee and approval was granted. Also, the consent was obtained from the volunteers included in the study.

### Statistical Analysis

SPSS 21 was used for statistical tests. Data were represented as mean ± standard deviation for numeric variables and as frequency and percentage for categorical variables. Normality of numeric variables was assessed by Shapiro-Wilk test. Comparisons of normally distributed variables were performed using t-tests, while the Mann-Whitney U test was used for nonnormally distributed nonparametric variables. The chi-square test was used to determine whether there was a relationship and dependency between two variables. Values of p<0.05 were considered statistically significant.

## Results

Eighty-five patients were included in the study. Bone marrow aspiration and biopsy were performed for 67 patients who required confirmation of the diagnosis of ITP. Among those, 49 were female and 36 were male. The median ages of the patients in the splenectomy and eltrombopag groups were 43.2 and 50.5 years, respectively. There were 38 patients in the splenectomy group and 47 patients in the eltrombopag group. There was no statistically significant difference between the two groups in terms of age, sex, comorbidities, bleeding score, WBC count, neutrophil and lymphocyte counts, hemoglobin, MCV, or platelet count. Characteristics of the treatment groups are summarized in Table 1.

Table 2 presents data on the time to achieve response (R) or complete response (CR) in the second line of treatment; R/CR at the 1^st^, 3^rd^, 6^th^, 12^th^, and 24^th^ months of second-line therapy; and the total duration of R/CR of the treatment groups. Time to R/CR was significantly shorter in the splenectomy group (p=0.001). The R/CR rate at the 1^st^ month was also significantly higher in the splenectomy group (p=0.023). However, R/CR rates at the 3^rd^, 6^th^, 12^th^, and 24^th^ months and total duration of R/CR did not exhibit statistically significant differences between the groups.

One patient in the splenectomy group developed deep vein thrombosis. In the eltrombopag group, one patient experienced headache, one patient had elevated liver enzymes, one patient developed bone marrow fibrosis, one patient developed chronic myelomonocytic leukemia (CMML), one patient developed basal cell carcinoma, and one patient developed pancreatic cancer. There was no statistically significant difference in adverse events between treatment groups (p=0.105). Bone marrow fibrosis was detected in the patient who underwent bone marrow biopsy 3 years after eltrombopag treatment because platelet increase could not be achieved despite increasing the dose of eltrombopag. The diagnosis of CMML was made 14 months after the patient started eltrombopag. A blast count below 5% was considered as CMML-0 and the patient was followed without treatment.

Patients receiving eltrombopag were stratified according to whether they received the treatment in the second or third line. Table 3 provides data on age and sex; comorbidities; bleeding score; WBC, neutrophil, and lymphocyte counts; hemoglobin, MCV, MPV, and platelet count; time to achieve R/CR; R/CR at the 1^st^, 3^rd^, 6^th^, 12^th^, and 24^th^ months of second-line treatment; and the total duration of R/CR. Patients who received eltrombopag in the third line had significantly higher WBC counts (p=0.002). On the other hand, no significant difference was observed between the groups in terms of age, sex, comorbidities, bleeding score, neutrophil and lymphocyte counts, hemoglobin, MCV, or platelet count. The R/CR rates at the 1^st^, 3^rd^, 6^th^, 12^th^, and 24^th^ months and the total duration of R/CR also did not exhibit statistically significant differences between the groups.

Eltrombopag treatment was discontinued with no tapering due to adverse effects or the patient’s refusal for various reasons in 20 patients after a median of 54.1 months (range: 1-151). Among them, 12 patients (60%) did not experience a loss of response. The median duration of eltrombopag treatment was 25.6 (range: 2-64) months and median follow-up was 67.9 (range: 20-151) months in these patients. The follow-up period after discontinuation of eltrombopag was 12.4 months, median platelet count was 159,000/mm^3^, and bleeding score was 0.0.

## Discussion

As highlighted in the updated international consensus report, initial treatments for patients with newly diagnosed ITP are usually corticosteroid-based regimens, intravenous immunoglobulin (IVIg), and anti-D. However, the most commonly preferred treatments are corticosteroids. IVIg can also be added to the treatment for patients with active bleeding, patients who are scheduled to undergo emergency interventions, or patients who experience adverse events with glucocorticoids, even though this approach is not standardized [16,17,18].

Because there are no randomized controlled studies that directly compare second-line treatment options in ITP, treatment decisions are mostly based on patient characteristics and clinical preferences. Second-line medical treatments supported by robust evidence are rituximab and TPO-RAs. Splenectomy is still also preferred as a surgical option today [18,19,20].

Splenectomy is among the second-line treatment options with the greatest likelihood of achieving long-term remission and changing the course of the disease [21]. Provan et al. [18] recommended waiting at least 12 months after the diagnosis to rule out possible spontaneous remission. As new drugs are available for ITP, splenectomy is often postponed. If the patient is working in a risky profession or has a thrombotic history or immunosuppression, splenectomy is considered as the second line. In developing countries, and especially for younger patients, it is still recommended for financial reasons and the difficulties in obtaining novel drugs. Kojouri et al. [22] conducted a systematic review of 47 case series including 2623 adult patients undergoing splenectomy and found rates of 66% complete response and 88% overall response. The mean duration of response was approximately 12 years in this patient group [22]. Platelet count often tends to rise within 1 to 2 days after splenectomy, while a delayed response can be observed at up to 8 weeks [23]. The only clinical parameter predicting splenectomy response is the patient’s age, with younger patients achieving higher response rates [22]. Even though there is no distinct age cut-off, splenectomy was recommended for patients younger than 50 years in two different studies [24,25]. In our study, the median age of the patients in the splenectomy group was 43.2 years. The higher median age of the patients in the eltrombopag group was due to elderly patients ineligible for splenectomy inevitably being in this treatment arm.

The most common complications after splenectomy, which is an irreversible procedure, are infections and venous thromboembolism. In the review by Kojouri et al. [22], complications occurred in 88 of 921 patients (9.6%) undergoing laparoscopic splenectomy and in 318 of 2465 patients (12.9%) undergoing open splenectomy. The risk of infection is highest in the early postoperative period. In another series of 3812 patients undergoing splenectomy for various indications, the rate of infections was 10.2% in the first 90 days [26]. However, this rate declines to 1%-3% in the long term [27]. In our study, no infectious side effects were found. The risk of VTE also increases after splenectomy. In the aforementioned cohort of 3812 patients undergoing splenectomy, the risk of VTE in the first year was reported to be 1.9%. Observed thromboses in that study were deep vein thrombosis in half of the patients, pulmonary embolism in a quarter, and portal or splenic vein thrombosis in the remaining quarter [26]. Considering these potential complications as well as the possibility of spontaneous remission in ITP, postponing splenectomy for 6 to 12 months after diagnosis may help avoid needless interventions. However, this duration may be shortened for symptomatic patients with severe thrombocytopenia [28]. In our study, response was observed at a mean of 1.9 days in 38 patients undergoing splenectomy and this response was durable for a mean of 43 months. One patient (2.6%) experienced deep vein thrombosis.

Studies assessing the efficiency of eltrombopag have reported overall response rates of about 80%, including temporary responses. The RAISE study, which randomized 197 adult patients into eltrombopag and placebo arms, showed that the mean platelet count of patients receiving eltrombopag was 74,000/mm^3^ and their response rate was 79%. This rate showed no difference among patients who underwent splenectomy and those who received other therapies prior to eltrombopag. The long-term response rate was 51% in patients with splenectomy and 66% in patients without splenectomy [29]. In another study with 110 adult patients, rates of response to eltrombopag and a placebo were found to be 59% and 16%, respectively [30]. The EXTEND study revealed that the 3-year response rates of 299 patients using eltrombopag were 80% for splenectomized and 89.3% for non-splenectomized patients [31]. In a recent study assessing real-life data, the researchers reported that rates of response to eltrombopag were not affected by factors including splenectomy status or initial platelet count. Six of those patients stopped eltrombopag after a median sustained response of 796 days and remained in remission for a median follow-up of 624 days [32]. Similarly, González-López et al. [33] reported that 51% of patients remained in remission in the 6^th^ month after eltrombopag cessation after sustained response.

Eltrombopag is usually well tolerated. The most common side effects are headache, gastrointestinal complaints, elevated liver enzymes, thrombosis, and bone marrow fibrosis. In the EXTEND study, 14% of patients had to cease medication due to adverse events. Among these, hepatobiliary adverse events were observed in 7 patients, cataract in 4 patients, deep vein thrombosis in 3 patients, cerebral infarction in 2 patients, headache in 2 patients, and myelofibrosis in 2 patients. The rate of thromboembolic events and hepatobiliary adverse events did not show any increase with 1 year of therapy [29,34]. In our study, median duration of response was 34.9 months in patients receiving eltrombopag and only 55% maintained response into the 2^nd^ year. There was no significant difference in the response rates of patients with and without a history of splenectomy prior to eltrombopag. Twelve of 20 patients who stopped eltrombopag treatment remained in remission. The observed adverse events were consistent with those reported in the literature; however, malignancy development in 3 (6.3%) patients was noteworthy. The EXTEND study did not reveal a significant difference between the treatment and placebo arms in terms of malignancies [34]. In a multicenter study from Turkey that reported the 12-month data of 40 patients on eltrombopag, there were also no eltrombopag-associated malignancies [35]. We could not identify a direct association between the observed malignancies and eltrombopag use, but we think that these malignancies may be incidental due to the higher median age in the eltrombopag group. Furthermore, a statistically significant difference was found in our study in terms of pretreatment leukocyte counts between administration of eltrombopag as second-line and as third-line treatment. This difference may be attributable to splenectomy, as the patients receiving third-line eltrombopag had previously undergone splenectomy.

## Conclusion

Comparing the splenectomy and eltrombopag arms of the present study, even though time to achieve response and the response rate at the 1^st^ month were in favor of splenectomy, this advantage disappeared when overall response rates and response rate at the 2^nd^ year were considered. However, more adverse events were observed in the eltrombopag group. Observed malignancies in 3 patients are inconsistent with the reported data in the literature. Using eltrombopag in the second or third line of therapy does not yield any difference in terms of time to achieve response. The overall response rate was higher when eltrombopag was used as the third-line treatment after splenectomy compared to second-line treatment, but this difference did not reach statistical significance. Randomized prospective studies comparing treatment options such as splenectomy, immunosuppressive therapies, and eltrombopag head-to-head are required for the standardization of second-line and more advanced therapies for immune thrombocytopenia.

## Figures and Tables

**Table 1:**
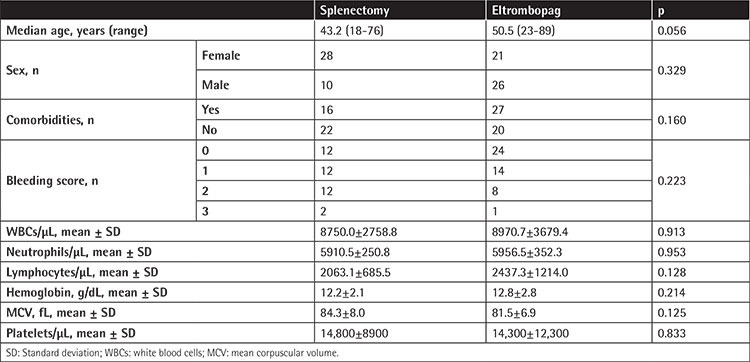
Patient characteristics.

**Table 2:**
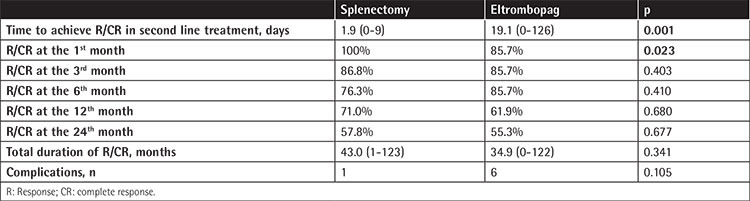
Comparison of treatment response between splenectomy and eltrombopag groups.

**Table 3:**
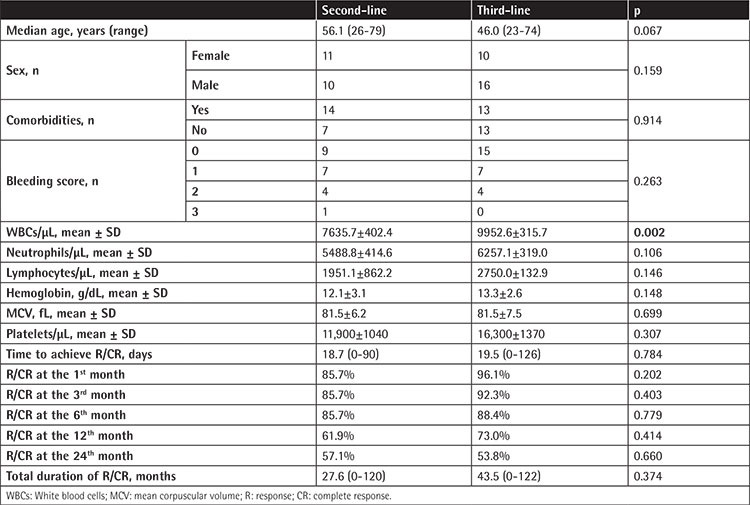
Eltrombopag as second-line or third-line treatment.
